# Psychological Safety, Job Crafting, and Employability: A Comparison Between Permanent and Temporary Workers

**DOI:** 10.3389/fpsyg.2019.00974

**Published:** 2019-05-01

**Authors:** Judith Plomp, Maria Tims, Svetlana N. Khapova, Paul G. W. Jansen, Arnold B. Bakker

**Affiliations:** ^1^School of Business and Economics, Vrije Universiteit Amsterdam, Amsterdam, Netherlands; ^2^Department of Psychology, Erasmus University Rotterdam, Rotterdam, Netherlands; ^3^Department of Psychology, University of Johannesburg, Johannesburg, South Africa

**Keywords:** employability, job crafting, psychological safety, permanent workers, temporary workers

## Abstract

Employability is one of the leading challenges of the contemporary organizational environment. While much is known about the positive effects of job crafting on employees’ employability in general, little is known about its effects when employment contacts are different. Differentiating between temporary and permanent workers, in this article we investigate how in the environment of psychological safety, these two types of employees engage in job crafting, and how job crafting is related to their perceived employability. Data were collected among two samples, consisting of temporary agency workers (*N* = 527), and permanent employees (*N* = 796). Structural equation modeling (SEM) analyses indicated a different pattern of results for the two groups: for permanent employees, increasing challenging job demands was positively, and decreasing hindering job demands was negatively related to perceived employability. Moreover, psychological safety was related to all job crafting dimensions. For agency workers, only increasing structural job resources was related to employability, while psychological safety was negatively associated with crafting hindrances. These findings suggest that a climate of psychological safety is particularly effective for permanent employees in fostering job crafting and employability.

## Introduction

The past decade has witnessed many significant changes in contractual arrangements between employers and workers. Nowadays, next to a broad pull of full-time workers employed on permanent contracts, there are increasingly more workers who work part-time and are employed on temporary contracts (Baruch and Altman, 2016; Katz and Krueger, 2016; Spreitzer et al., 2017). These latter workers can be associated with agencies that find assignments for them, or they can be contracted as freelancers (Cappelli and Keller, 2013). Central to these new employment relationships is workers’ external marketability (Arthur et al., 2017), which is often referred to as perceived employability in the work and organization literature (Vanhercke et al., 2014). Perceived employability can be defined as one’s perceived possibilities to obtain and maintain employment, and it assumes that workers are increasingly responsible for their own work and career development (De Vos and Van der Heijden, 2017). While many scholars call for workers’ career responsibility, very few studies actually address what it means to take such responsibility, and more specifically, what it entails when we refer to different types of workers.

In this study we propose that, in terms of employability, temporary, and permanent workers can benefit from job crafting activities – i.e., make proactive changes in one’s job tasks or relationships (Wrzesniewski and Dutton, 2001). For instance, by actively seeking out ways to obtain feedback and gain job-related skills, or utilize developmental possibilities within their organization, workers may prepare themselves for professional growth, and future work. Earlier studies have shown a positive relationship between job crafting and employability among samples of permanent employees (Tims et al., 2012; Brenninkmeijer and Hekkert-Koning, 2015). However, the current literature provides no further insight in how the temporary workforce applies job crafting as a strategy to improve their employability, even though being employable is even more urgent for this group of workers compared to others (De Cuyper et al., 2009). To illustrate, research has shown that temporary workers are often easily transferred and replaced (Kalleberg, 2000; Connelly and Gallagher, 2004; De Cuyper et al., 2009) and have little access to organizational development programs and training opportunities (Forrier and Sels, 2003; De Cuyper et al., 2009). Therefore, in this article we examine how temporary workers, in comparison with permanent employees, can use job crafting to develop skills and competencies needed to become and stay employable.

Although job crafting refers to self-initiated actions, these initiatives cannot be seen independently from their organizational context. Hence, it is likely that some situations provoke more proactive behaviors than others (Parker et al., 2006; Van Wingerden and Poell, 2017). Since career responsibility is shifting toward the individual, organizations could play a leading role in creating a work environment that enables employees to craft their job. Furthermore, employee expectations regarding the consequences of their initiatives are an important predictor of actual risk-taking and proactive behaviors (Crant, 2000). We therefore take into account to what extent workers feel they can make changes in their work without negative consequences, and include psychological safety as a contextual correlate of job crafting (Baer and Frese, 2003).

The present study extends previous research in three important ways. First, job crafting has primarily been studied among permanent employees, whereas nowadays, alternative work arrangements are increasingly more common. By testing our research model in a sample of both permanent employees and temporary agency workers, we examine how job crafting behaviors relate to individuals’ perceived chances to gain new employment, and to what extent these proactive behaviors differ between workers with different contract arrangements. We therefore aim to contribute to a better understanding of proactive employee behavior in the context of temporary work. Second, by taking into account psychological safety as an organizational facilitator of job crafting, we respond to the call for more research on the organizational conditions encouraging employees to actively craft their job (Crant, 2000; Oldham and Fried, 2016). Third, we contribute to the literatures on both proactive employee behaviors and career studies by linking job-related proactive behaviors to a career-related outcome. So far, studies have mainly focused on job-related outcomes of proactive work behavior such as, job satisfaction, work engagement, and job performance (Rudolph et al., 2017), whereas we extend recent research (e.g., Akkermans and Tims, 2017) by investigating how job crafting is related to individuals’ perceived chances on the labor market.

## Theoretical Background

### Psychological Safety and Job Crafting

#### Conceptualization

Psychological safety refers to employee perceptions regarding the consequences of interpersonal risk-taking (Edmondson, 1999; Baer and Frese, 2003). Psychological safety can be studied as an individual, team, or organizational phenomenon (Edmondson and Lei, 2014). We follow the argumentation by Kark and Carmeli (2009), who stated that individual perceptions of the work environment are likely to result in individual behavioral outcomes, especially with regard to risk-taking. Edmondson (2004) argued that individuals who feel psychologically safe, are more likely to engage in voice behaviors, initiative taking, and proactive behaviors. Indeed, psychological safety has been linked to speaking up (i.e., voice; Detert and Burris, 2007), and has been found a key factor in learning and creativity at work (Edmondson, 1999; Kark and Carmeli, 2009). Due to the often uncertain nature of these actions and possible risks of failure, an environment in which employees feel confident to challenge the status quo without fear of negative consequences is paramount in the display of proactive work behaviors.

A specific form of proactivity at the job-level is job crafting. Job crafters engage in self-initiated actions aimed at modifying their job tasks and/or relationships to create a better fit with their personal needs, goals, and preferences (Wrzesniewski and Dutton, 2001; Tims and Bakker, 2010). Tims and Bakker (2010) used job demands-resources (JD-R) theory (Demerouti et al., 2001; Bakker and Demerouti, 2014) as a framework to conceptualize job crafting. Accordingly, all job characteristics can be classified as either job demands or job resources. Job demands (e.g., workload and role ambiguity) refer to aspects of the job that require physical or mental effort from the employee. Job resources (e.g., feedback, autonomy, and support) are job characteristics that help employees deal with hindering job demands and provide opportunities for individual development. In line with Tims et al. (2012) and based on the JD-R theory, we operationalize job crafting using four dimensions. First, increasing structural job resources refers to accumulating job resources by mobilizing autonomy, variety, and developmental opportunities. Second, employees increase their social job resources by seeking out social support and feedback from their coworkers or supervisors. Third, by increasing challenging job demands, whereby one pursues extra job tasks or initiates new projects, employees are able to make their job more stimulating (Harju et al., 2016). Finally, decreasing hindering job demands refers to reducing emotional or cognitive job demands. When employees feel systematically overwhelmed by hindering cognitive or emotional strains, they may proactively lower these job hindrances (Tims et al., 2012).

### Relationship Between Psychological Safety and the Job Crafting Dimensions

Given that proactive behaviors are self-initiated, Grant and Ashford (2008) argued that there are ego and image risks associated with these actions, because failure cannot be blamed on external circumstances. This notion is emphasized by expectancy theory, which states that individuals carefully take into account the anticipated costs and benefits of their actions, before they challenge the status quo (Vroom, 1964, 2005; Morrison and Phelps, 1999). We reason that both temporary and permanent employees, who experience their work environment as a safe place to initiate change, will be inclined to engage in job crafting.

First, we expect perceptions of safety to relate to increasing structural job resources, characterized by trying to develop professional skills and engaging in new learning experiences. Considering that crafting structural job resources consists of learning-related initiatives, including broadening one’s skill set, we argue that employees who have no concern about the reaction from others are also not afraid of possible embarrassment and status loss often linked to these initiatives (Milliken et al., 2003), resulting in the crafting of more structural job resources. Additionally, research has shown that people who feel psychologically safe and do not fear negative consequences concerning their self-image, status or career, are likely to take more interpersonal risks, including actively asking questions, and seeking feedback from either colleagues or supervisors (Edmondson, 1999; Carmeli et al., 2009). Accordingly, we expect employees who feel psychologically safe, also engage in job crafting behaviors aimed at increasing social job resources.

Turning to crafting job demands, we expect that employees who feel safe to take risks, will be more motivated to initiate work projects and take on new tasks, and as such engage in crafting challenging job demands. Similarly, when an employee wants to start a new and challenging project, but expects a backlash from the manager when this projects fails to meet the anticipated outcomes, it is likely that they will refrain from engaging in this undertaking. Especially reducing hindering job demands may be perceived as risky crafting behavior because of the potentially negative consequences this has for the work of others and the relationships with clients, colleagues, and managers (cf. Tims et al., 2015). For instance, in a situation where employees feel that they have to refrain from certain work tasks in order to deal with the work pressure, it is important that the employee perceives the work environment supportive of these actions (i.e., feel psychologically safe), before they will actually decrease these hindering job demands.

Similarly, learning-related initiatives (i.e., crafting structural job resources), as well as actively reaching out to co-workers (crafting social job resources) can both be perceived as possible risky behaviors and can provoke an unwanted response from coworkers and/or managers (Frese and Fay, 2001; Parker et al., 2006). Employees who feel that they can engage in risk-taking behavior, including crafting one’s job resources, without negative fall-out from within their direct organizational environment, will be more inclined to actually craft their job.

We further argue that psychological safety is equally important for temporary and permanent workers related to job crafting. For employees who hold a permanent position, engaging in job crafting could have far-reaching consequences regarding the future of their job, status, and development within their company. To illustrate, when permanent employees expect a certain response following their job crafting initiatives, they may anticipate to be taken into consideration for a promotion or not, to be negatively or positively appraised by colleagues and managers, or to get more or fewer opportunities to participate in projects. Therefore, their perception whether the organizational environment is a safe place to engage in risk-taking behaviors, is likely to be related to job crafting initiatives. For temporary workers, who are relatively new in an organization and only have a limited timeframe to perform, their position may come with uncertainty regarding what is expected from them and how the organization operates. As such, feeling psychologically safe could help temporary workers overcome these insecurities and could make them feel free to craft their jobs, which allows them to get familiar with the organization. Hence, we expect similar relationships between psychological safety and job crafting behaviors for both temporary and permanent employees.

Hypothesis 1: For permanent employees, psychological safety is positively related to (a) increasing structural job resources, (b) increasing social job resources, (c) increasing challenging job demands, and (d) decreasing hindering job demands.

Hypothesis 2: For temporary employees, psychological safety is positively related to (a) increasing structural job resources, (b) increasing social job resources, (c) increasing challenging job demands, and (d) decreasing hindering job demands.

### Job Crafting and Employability

In this study, we focus on external employability, defined as the degree to which employees perceive their ability to move between organizations (De Cuyper et al., 2012). We are particularly interested in subjective employability, because objective employability evaluations are not likely to account for contextual factors, including sector-specific labor market information (Silla et al., 2009). Therefore, an individual’s appraisal of employability may contain a more comprehensive assessment of one’s chances to obtain new employment. Moreover, we are interested in external employability, because it is important for both temporary and permanent workers to be able to secure work outside of the current organization due to the fast-paced changes and insecurity in the current labor market (De Cuyper et al., 2012). Given the increased emphasis on the worker’s own responsibility for securing work (Van Der Heijde and Van Der Heijden, 2006; Fugate and Kinicki, 2008), job crafting may be a relevant on-the-job proactive behavior that allows workers to become or stay externally employable.

By crafting job resources and demands employees create a work environment that provides opportunities beneficial and relevant to their current, as well as prospective career progress. The accumulation of job resources and challenges through job crafting likely stimulates the development of relevant knowledge, skills, and relationships that help employees be adaptable and facilitate personal growth. Research has indeed shown that proactive initiatives at the job level relate to both work- and career-related outcomes (Plomp et al., 2016; Akkermans and Tims, 2017). Moreover, employees who take initiative with regard to professional self-development, are willing to consider more and different job opportunities, which is related to perceived external employability (Wittekind et al., 2010). These findings are supported by Akkermans and Tims (2017), who showed that job crafting was positively related to perceived internal and external employability, indicating that the expansion of job resources and/or demands stimulates personal development and the ability to cope with change. In light of these results, to become and stay employable, employees need to actively seek out ways to develop their current skill set, as well as seek out feedback, and advice from their professional network. Studies so far have mainly focused on permanent employees and have not differentiated between types of workers. However, in terms of feeling employable, we expect different relationships concerning job crafting behaviors for temporary and permanent workers.

For permanent employees, the increased responsibility, professional experience, and networking opportunities gained by crafting structural and social job resources, as well as the experience of engaging in new initiatives (i.e., increasing challenging job demands), is likely to be seen as transferable to other jobs and organizations, thereby contributing to feelings of employability. Accordingly, Van Emmerik et al. (2012) showed that employees who encountered more resources in their job, also experienced more external job opportunities, and as a result felt more employable. These findings suggest that employees who seek feedback, ways to develop themselves, and new challenges, accumulate a greater pool of job resources, enabling them to learn and develop relevant professional skills, and in turn increasing their perceptions of employability. Additionally, Brenninkmeijer and Hekkert-Koning (2015) found that increasing structural and social job resources, as well as challenging job demands related to higher levels of employability. In contrast, permanent employees who lower their hindrances at work may feel that their qualities are not in line with the demands of their job and may try to make the job more manageable for themselves (Tims et al., 2012; Brenninkmeijer and Hekkert-Koning, 2015). When decreasing hindering demands, workers may feel that they lack competences and skills needed to perform, which reduces their perceived competitiveness on the labor market.

For temporary employees, we expect that particularly the experience of crafting structural job resources will be viewed as transferable to new jobs and will contribute to their perception of being employable outside of their current organization. By increasing structural job resources, temporary employees are able to enhance their overall skill-set and professional abilities, which is highly useful for securing a new job or obtaining a permanent position. In contrast, we argue that crafting social job resources and job demands are less likely to be evaluated as transferable to external jobs. Regarding social job resources, it seems unlikely that seeking job-related feedback and advice will be seen as transferable, because temporary workers often do not know where they will be employed next and whether this job-specific guidance will help them in a different organizational setting. In addition, we expect that alterations in job demands are unrelated to feelings of employability. We follow the reasoning of Hakanen et al. (2006), who stated that a work environment consists of “givens” (i.e., work characteristics that are relatively stable over time and difficult to change) and “alterables” (i.e., aspects of the organizational environment that can be influenced on the short-term). Because changes in job resources are more easily accomplished on the short-term, this is also achievable for temporary workers who stay within an organization for a limited amount of time. However, to change job demands, including workload and job requirements, more time and effort is needed, which makes it difficult for temporary workers to modify (Tims et al., 2015). Moreover, making investments that take time and effort are likely to reap benefits on the long term, and quite possibly only after the temporary worker has left the organization. As such, effort needed to craft challenging job demands is likely to distract temporary workers from their core job tasks and performance, and is not likely to contribute to a greater sense of employability. Particularly, decreasing hindering job demands may contribute to a declining sense of employability. Comparable to permanent employees, lowering one’s hindrances, may function an indicator for workers that they are unable to deal with the requirements of their current job, making them feel less employable for other future jobs.

Hypothesis 3: For permanent employees (a) increasing structural job resources, (b) increasing social job resources, and (c) increasing challenging job demands are positively related to perceived external employability, whereas (d) decreasing hindering job demands is negatively related to perceived external employability.

Hypothesis 4: For temporary workers, the relationship between (a) increasing structural job resources and perceived external employability are positively related to perceived employability, whereas (b) decreasing hindering job demands is negatively related to perceived external employability.

### Indirect Effect of Psychological Safety on Employability Through Job Crafting

We suggest an indirect relationship between psychological safety and perceived employability through job crafting. Work environments where permanent and temporary employees feel psychologically safe, are likely to support employees in feeling sufficiently comfortable and confident to actively adjust job demands and resources according to their personal needs and preferences.

In turn, through crafting structural job resources and challenging job demands, permanent employees may be able to shape their work environment so that they have access to job resources that build their skill set and enhance their performance on the job, while also gaining skills, and abilities that may be relevant for other jobs. In addition, by crafting social job resources these workers may broaden and improve their job-related knowledge, which may inform them of their chances of similar employment elsewhere. However, we expect a different indirect relationship between psychological safety and employability via decreasing hindering job demands. On the one hand, permanent employees who feel safe to craft, may also be inclined to reduce aspects of the job that are considered a hindrance. However, by lowering their job demands, workers may experience that their skills are not sufficient to perform well within their job (Gordon et al., 2015), and feel that their abilities are not that appealing to other organizations.

Turning to temporary workers, we argue that in particular crafting structural job resources relates to perceived employability. By crafting job resources aimed at obtaining and developing relevant professional competencies, it is likely that temporary workers perceive themselves as able to secure future work, and feel more employable. Comparable to permanent employees, decreasing hindrances at work may function as a signal that they are not skilled enough for the current job and as such increases the feeling of being less employable.

Hypothesis 5: For permanent employees, psychological safety is positively related to perceived external employability through (a) increasing structural job resources, (b) increasing social job resources, and (c), increasing challenging job demands. Additionally, psychological safety is positively related to (d) decreasing hindering job demands, which is in turn negatively related to perceived external employability.

Hypothesis 6: For temporary employees, psychological safety is positively related to perceived external employability through (a) increasing structural job resources. Additionally, psychological safety is positively related to (b) decreasing hindering job demands, which is in turn negatively related to perceived external employability.

## Materials and Methods

### Participants and Procedure

We recruited a sample consisting of temporary agency workers and a sample of permanent workers. The sample of permanent employees (*N* = 796) was recruited via students participating in a quantitative research methods course at a large Dutch university. In this sample, 54.80% was female and the mean age was 40.67 years (*SD* = 13.31). Participants reported an average job tenure of 10.06 years (*SD* = 9.43) and their mean work experience was 19.34 years (*SD* = 12.83). These employees were also active in various organizational industries, including corporate and financial services (21.3%), healthcare and social services (15.1%), and government institutions (9%). The sample of agency workers (*N* = 527) was recruited via one of the largest temporary staffing agencies in Netherlands. In this sample, 56.40% was female and their mean age was 41.85 years (*SD* = 13.94). Their average job tenure was 2.71 years (*SD* = 5.38) and their mean work experience was 19.86 years (*SD* = 14.14). The agency workers were active in various organizational sectors, such as transport (13%), government institutions (12.5%), and production (10.8%).

All participants were required to work at least 3 days per week to be eligible for the study. Employees received an e-mail with information about the aim of the study, a link to the online questionnaire, and an explanation of the confidentiality afforded to the participants. The survey contained a consent form including all relevant information before the launch of the survey. Informed consent was obtained to ensure that the researchers had the right to use the collected data. The questionnaire did not include any sensitive, personal privacy, ethical and/or moral themes. The survey is available and can be provided upon a request. Moreover, data for this study was collected in 2016 and the ethics approval was not required at the time as per the university’s guidelines and national regulations. However, this study was conducted in accordance with the Research Ethics and Regulations of the School of Business and Economics of the Vrije Universiteit Amsterdam. The online survey was anonymous and informed consent was obtained from all participants that were approached to take part in the survey.

Temporary agency workers did not differ significantly from permanent employees in terms of the demographic variables age [*t*(1321) = 1.54, *p* = 0.75] and work experience [*t*(1321) = 0.69, *p* = 0.07]. Temporary agency workers had on average a significantly lower job tenure compared to permanent employees [*t*(1185) = −15.72, *p* < 0.001]. This difference can be explained the inherent short contract duration when working via a staffing agency. Furthermore, we tested for mean differences on the study variables between the group of agency workers and permanent employees by conducting an independent samples *t*-test in SPSS. Table 1 shows the results of these analyses. Interestingly, temporary agency workers report on average significantly lower scores on all study variables compared to permanent employees, with the exception of increasing structural job resources. We elaborate on these findings in the discussion.

**TABLE 1:** Results of *t*-tests comparing temporary agency workers and permanent employees on the study variables.

	**Temporary agency**		**Permanent**		***t*-test**
	**workers**		**employees**		
	***M***	***SD***	***M***	***SD***	
Psychological safety	3.57	0.66	3.66	0.61	−2.42**
Increasing structural job resources	3.46	0.92	3.55	0.86	−1.70
Increasing social job resources	2.44	0.75	2.60	0.78	−3.58**
Increasing challenging job demands	2.59	0.85	2.90	0.80	−6.74**
Decreasing hindering job demands	1.89	0.71	2.02	0.68	−3.37**
Perceived employability	3.33	0.97	3.49	0.89	−3.16**

**p < 0.05.

### Measures

*Psychological safety* was measured with five items of Edmondson’s (1999) psychological safety scale. Example items are: “In our company, one is free to take risks,” and “As an employee in our company, one is able to bring up problems and tough issues.” A five-point scale was used with response categories ranging from 1 (strongly disagree) to 5 (strongly agree). Cronbach’s *α* for this measure was 0.67 in the sample of temporary agency workers and 0.65 in the sample of permanent employees.

*Job crafting* was measured with the 21-item job crafting scale developed by Tims et al. (2012). Increasing structural job resources was measured with five items (e.g., “I try to develop my capabilities,” *α* = 0.78 for temporary agency workers and 0.83 for permanent employees), as well as increasing social job resources (e.g., “I ask colleagues for advice,” *α* = 0.77 for temporary agency workers and 0.80 for permanent employees). The other two dimensions this study were “increasing challenging job demands” (consisting of five items, e.g., “If there are new developments, I am one of the first to learn about them and try them out”; *α* = 0.79 for temporary agency workers and 0.77 for permanent employees), and “decreasing hindering job demands” (consisting of six items, e.g., “I try to ensure that my work is emotionally less intense”; *α* = 0.81 for temporary agency workers and 0.80 for permanent employees). Respondents indicated how often they engaged in each of the behaviors on a 5-point Likert scale ranging from 1 (never) to 5 (very often).

*Perceived external employability* was measured with three items developed by Janssens et al. (2003). An example item is “I am confident that I would find another job if I started searching.” A five-point scale was used with response categories ranging from 1 (strongly disagree) to 5 (strongly agree). Cronbach’s *α* was 0.81 for temporary workers and 0.82 for permanent employees.

### Strategy of Analysis

First, the measurement model was evaluated using confirmatory factor analysis (CFA). Latent variables were modeled with scale items (i.e., psychological safety, increasing structural job resources, increasing social job resources, increasing challenging demands, decreasing hindering demands, and perceived employability). The following fit indices were used to evaluate model fit: the comparative fit index (CFI), the Tucker-Lewis index (TLI), and the root mean square error of approximation (RMSEA). With CFI and TLI values above 0.95, and RMSEA below 0.06, model fit is good, and CFI and TLI values above 0.90 and RMSEA below 0.08 are adequate (Hoyle, 1995; Hu and Bentler, 1999).

Second, the proposed research model was tested using structural equation modeling (SEM) with the AMOS software package (Arbuckle, 2005). We tested our structural model in both samples (i.e., permanent employees and temporary agency workers). In addition, we conducted a multigroup SEM analysis, which allows testing of one model in the two contract groups simultaneously (Byrne, 2004) and examines the differences in structural pathways in both groups of employees. To estimate and test the specific indirect effects we applied the phantom model approach (Macho and Ledermann, 2011), which allows to assess the indirect effect while also taking into account the other indirect pathways in the model. As such, we can test for multiple specific indirect effects separately, instead of receiving a single estimate for the indirect effect of the model as a whole. By means of duplicating a specific indirect relationship, consisting of the latent variables representing that specific relationship (e.g., the specific effect of psychological safety on perceived employability via crafting structural job resources), we are able to create a phantom model. Moreover, the parameters of the latent phantom variables are constrained to the path values in the original SEM model, meaning that these do not influence the estimation of the SEM model, and as such provides an estimation of the specific effects (Macho and Ledermann, 2011).

## Results

### Descriptive Statistics

The descriptive statistics, including the means, standard deviations, and correlations of the study variables for both permanent employees and temporary agency workers can be found in Tables 2, 3. Occupational level was positively and significantly correlated with perceived employability. In addition, both work experience and age were negatively correlated with all job crafting dimensions, as well as perceived employability. As can be expected, work experience and age correlated very strongly (*r* = 0.94 for permanent employees and *r* = 0.90 for temporary agency workers). Therefore, to prevent multicollinearity, we only controlled for work experience and occupational level in our analyses.

**TABLE 2:** Descriptive statistics and inter-correlations of the study variables for permanent employees, *N* = 796.

	***M***	***SD***	**1**	**2**	**3**	**4**	**5**	**6**	**7**	**8**	**9**	**10**
1. Age	40.67	13.31	–									
2. Job level	4.66	1.25	$0.09^{*}$	–								
3. Job tenure	10.06	9.43	$0.60^{**}$	-0.03	–							
4. Work experience	19.34	12.83	$0.94^{**}$	-0.03	$0.64^{**}$	–						
5. Psychological safety	3.66	0.61	0.05	$0.16^{**}$	0.04	0.03	–					
6. Increasing structural job resources	3.55	0.87	$-0.19^{**}$	$0.30^{**}$	$-0.19^{**}$	$-0.22^{**}$	$0.21^{**}$	–				
7. Increasing social job resources	2.40	0.91	$-0.31^{**}$	$0.25^{**}$	$-0.26^{**}$	$-0.33^{**}$	$0.15^{**}$	$0.46^{**}$	–			
8. Increasing challenging job demands	2.90	0.80	$-0.10^{**}$	$0.30^{**}$	$-0.17^{**}$	$-0.13^{**}$	$0.13^{**}$	$0.62^{**}$	$0.49^{**}$	–		
9. Decreasing hindering job demands	4.83	0.69	$-0.25^{**}$	0.03	$-0.21^{**}$	$-0.28^{**}$	$-0.15^{**}$	$0.11^{**}$	$0.36^{**}$	$0.23^{**}$	–	
10. Perceived employability	3.49	0.89	$-0.37^{**}$	$0.08^{*}$	$-0.34^{**}$	$-0.37^{**}$	$0.10^{**}$	$0.27^{**}$	$0.19^{**}$	$0.23^{**}$	0.02	–

*n* = 796. *p < 0.05, **p < 0.01.

**TABLE 3:** Descriptive statistics and inter-correlations of the study variables for temporary agency workers, *N* = 527.

	***M***	***SD***	**1**	**2**	**3**	**4**	**5**	**6**	**7**	**8**	**9**	**10**
1. Age	41.85	13.94	–									
2. Job level	3.72	1.26	$-0.28^{**}$	–								
3. Job tenure	2.71	5.38	$0.09^{*}$	-0.02	–							
4. Work experience	19.86	14.14	$0.90^{**}$	$-0.28^{**}$	0.08	–						
5. Psychological safety	3.35	0.61	0.01	$0.15^{**}$	0.03	0.01	–					
6. Increasing structural job resources	3.46	0.91	$-0.13^{**}$	$0.15^{**}$	0.03	$-0.15^{**}$	$0.10^{*}$	–				
7. Increasing social job resources	2.44	0.75	$-0.25^{**}$	$0.21^{**}$	$-0.10^{*}$	$-0.26^{**}$	$0.15^{**}$	$0.45^{**}$	–			
8. Increasing challenging job demands	2.59	0.85	$-0.20^{**}$	$0.17^{**}$	0.02	$-0.20^{**}$	0.05	$0.57^{**}$	$0.54^{**}$	–		
9. Decreasing hindering job demands	1.89	0.71	$-0.25^{**}$	0.06	-0.04	$-0.27^{**}$	$-0.13^{**}$	0.07	$0.31^{**}$	$0.27^{**}$	–	
10. Perceived employability	3.33	0.97	$-0.23^{**}$	0.03	-0.06	$-0.20^{**}$	-0.01	$0.18^{**}$	0.07	$0.12^{**}$	0.08	–

*n* = 527. *p < 0.05, **p < 0.01.

### Measurement Model and Invariance Test

The measurement model included six latent variables with the items as indicators of the latent factor (i.e., psychological safety, increasing structural job resources, increasing social job resources, increasing challenging job demands, decreasing hindering job demands, and perceived employability). In the sample of permanent employees, the measurement model showed a reasonable, albeit not perfect fit to the data: χ^2^ = 1114.794, *df* = 362, CFI = 0.90, TLI = 0.88, and RMSEA = 0.05. All factor loadings were significant and loaded substantially on their respective factor. Factor loadings ranged from 0.51 to 0.86 (all *p*’s < 0.001). After covariations were included – only between items belonging to the same dimension (i.e., crafting social job resources, challenging, and hindering job demands) theoretical construct (i.e., psychological safety) (cf. Byrne, 2005) – the measurement model showed a substantially better fit to the data: χ^2^ = 698.599, *df* = 296, CFI = 0.94, TLI = 0.93, and RMSEA = 0.04. In the sample of temporary agency workers, the measurement model showed a reasonable, albeit not perfect fit to the data: χ^2^ = 8390.823, *df* = 309, CFI = 0.88, TLI = 0.87, and RMSEA = 0.06. All factor loadings were significant and loaded substantially on their respective factor. Factor loadings ranged from 0.43 to 0.85 (all *p*’s < 0.001). After covariations were included - only between items belonging to the same dimension or theoretical construct (cf. Byrne, 2005) – the measurement model showed a substantially better fit to the data: χ^2^ = 618.572, *df* = 296, CFI = 0.93, TLI = 0.91, and RMSEA = 0.04. In addition, we tested whether the measurement model was invariant across agency workers and permanent employees. Model fit did not change substantially when factor loadings were constrained to be equal in both groups (Δχ^2^ = 39.90, Δ*df* = 27, *p* = 0.05). The factor structure nearly reached statistical significance, indicating that overall, the same underlying constructs were measured and the factor structure is largely equivalent in both groups (Byrne, 2004).

### Testing the Hypothesized Model for Permanent Employees

In hypotheses 1a–c, we proposed a positive relationship between psychological safety and increasing structural and social job resources, as well as challenging job demands. Our data showed a positive and significant relationship between psychological safety and (a) increasing structural job resources (β = 0.26, *p* < 0.001), (b) increasing social job resources (β = 0.19, *p* < 0.001), and (c) increasing challenging job demands (β = 0.16, *p* < 0.01). As such, we found support for hypotheses 1a–c. However, contrary to our expectation, the data showed a significant and negative relationship between psychological safety and decreasing hindering job demands (β = −0.19, *p* < 0.001). Thereby, hypothesis 1d was not confirmed.

Before elaborating on hypotheses 2a–d, which focus on temporary workers, we first discuss all hypotheses on permanent employees. As such, turning to hypothesis 3a, in which we proposed a positive relationship between increasing structural job resources and perceived employability, although in the expected direction, we found a non-significant relationship (β = 0.13, *p* = 0.18). In addition, hypothesis 3b, in which we predicted a positive relationship between increasing social job resources and perceived employability, was not supported by our results (β = −0.08, *p* = 0.33). However, we found a positive and significant relationship between increasing challenging demands and perceived employability (β = 0.24, *p* = 0.02), thereby confirming hypothesis 3c. Hypothesis 3d, which proposed a negative relation between decreasing hindering job demands and perceived employability, was also supported by our data (β = −0.15, *p* < 0.001).

**FIGURE 1:**
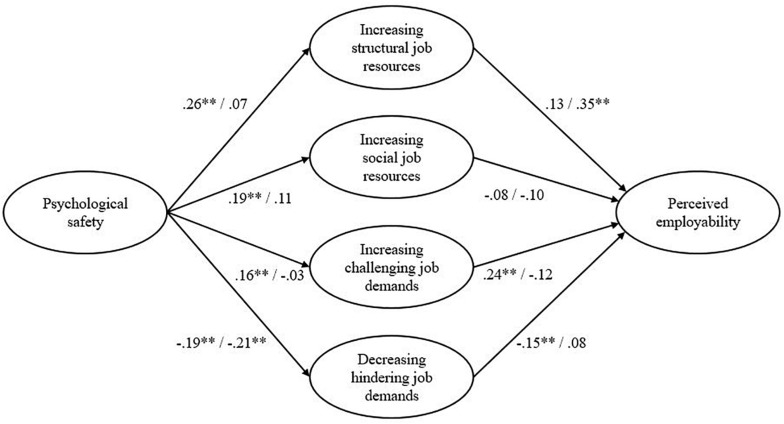
Results for structural equation modeling. Before slash: permanent workers (*N* = 796) and after slash: temporary workers (*N* = 527). **p < 0.001.

Last, we proposed a positive indirect relationship of psychological safety with employability, through increasing structural job resources. We used phantom models (Macho and Ledermann, 2011) to test the estimate of the specific indirect effects. The results showed that this indirect effect of increasing structural job resources was indeed significant (estimate = 0.12, *p* = 0.03). The bias-corrected confidence interval (B-CCI) ranged from 0.05 to 0.28. As such, hypothesis 5a was supported by our data. Additionally, we expected a similar indirect effect for increasing social job resources. The specific effect of increasing structural job resources in the relation between psychological safety and perceived employability was not significant (estimate = −0.03, *p* = 0.49) and the B-CCI ranged from −0.03 to 0.12), thereby hypothesis 5b was not confirmed. Turning to the indirect effect of increasing challenging job resources in the relationship between psychological safety and perceived employability, Phantom model analyses showed that this was also not significant (estimate = 0.03, *p* = 0.41) and the B-CCI ranged from −0.04 to 0.11). Therefore, hypothesis 5c was not supported. Last, we proposed that psychological safety was positively related to decreasing hindering job demands, which was in turn negatively related to perceived employability. The specific effect of decreasing hindering job demands in the relation between psychological safety and perceived employability was also not significant (estimate = 0.15, *p* = 0.36) and the B-CCI ranged from −0.01 to 0.06). Thereby, hypothesis 5d was also not confirmed. Overall, we found that for permanent employees, psychological safety was positively related to crafting structural and social job resources, as well as challenging job demands, and negatively related to decreasing hindering job demands. Additionally, increasing challenging job demands was positively, and decreasing hindering job demands was related negatively to perceived employability. Our hypothesized model, as depicted in Figure 1, showed an adequate fit to the data: χ^2^ = 838.782, *df* = 341, CFI = 0.93, TLI = 0.92, and RMSEA = 0.04.

### Testing the Hypothesized Model for Temporary Agency Workers

Regarding the psychological safety – job crafting relationships for temporary agency workers, the data showed that psychological safety was not significantly related to increasing structural job resources (β = 0.07, *p* = 0.23), and also not to increasing social job resources (β = 0.11, *p* = 0.08). Additionally, we found no significant relationship between psychological safety and increasing challenging job demands (β = −0.03, *p* = 0.64). However, psychological safety was significantly and negatively related to decreasing hindering job demands (β = −0.21, *p* < 0.01). As such, hypotheses 2a–d were not confirmed.

Furthermore, the data showed that for temporary agency workers, job crafting in the form of increasing structural job resources was positively and significantly linked to perceived employability (β = 0.35, *p* < 0.01), thereby confirming hypothesis 4a. However, decreasing hindering job demands (β = 0.08, *p* = 0.20) was not significantly related to perceived employability, which is not in support of hypothesis 4b.

Turning to the indirect relationships of psychological safety with employability through crafting structural job resources, our results showed that this effect was not significant (estimate = 0.04, *p* = 0.22). The B-CCI ranged from −0.01 to 0.15. Last, the specific effect of decreasing hindering job demands in the relation between psychological safety and perceived employability was significant (estimate = 0.05, *p* = 0.01) and the B-CCI ranged from −0.15 to −0.01). To conclude, for temporary workers, psychological safety was unrelated to all job crafting dimensions, with exception of decreasing hindering job demands. In addition, only increasing structural job resources was positively related to perceived employability. Overall, the model showed an adequate fit to the data: χ^2^ = 702.160, *df* = 341, CFI = 0.92, TLI = 0.91, and RMSEA = 0.05.

### Structural Invariance Test

To test whether our structural model was invariant across groups, we compared the fit of our hypothesized model to a model in which we constrained all factor loadings, path coefficients, and covariances to be equal across both groups (Byrne, 2016). The results of the multigroup analyses showed that the model fit of our freely estimated model (χ^2^ = 1541.999, *df* = 682, CFI = 0.93, TLI = 0.92, and RMSEA = 0.03), was significantly better compared to the constrained model (χ^2^ = 1797.304, *df* = 748, CFI = 0.91, TLI = 0.91, and RMSEA = 0.03; Δχ^2^ = 256.305, Δ*df* = 66, *p* < 0.001), meaning that the groups are different at the model level. To investigate whether the pathways in both groups of employees obtained contrasting results and were actually significantly different at the structural level, we constrained all structural pathways in our hypothesized model except the pathways that were significant in one of the groups and not in the other and conducted a chi-square difference test. After doing this, we found support for the idea that temporary workers indeed differed significantly from permanent employees on these structural paths (χ^2^ = 1557.025, *df* = 688, CFI = 0.93, TLI = 0.92, and RMSEA = 0.03, Δχ^2^ = 15.026, Δdf = 6, *p* = 0.01), mainly explained by the different pathways between psychological safety and job crafting.

### Additional Analyses

An alternative model was tested to examine a different potential research model, in which perceived employability relates indirectly to job crafting, through psychological safety. A possible rationale for this alternative model could be that individuals who believe that they are able to secure future work also feel more psychologically safe in their current job to make changes and engage in potential risky behaviors, such as job crafting, because they are less dependent on their current employer. On the other hand, those who feel that they would face difficulties finding another job may avoid any initiative-taking that could potentially harm their status and position within the company. As shown in Table 4, our original research model (M1), in which we proposed an indirect effect between psychological safety and perceived employability through job crafting behaviors, provided the best overall fit compared with our alternative model (M2) in both the sample of permanent employees (χ^2^ = 976.750, *df* = 344, CFI = 0.92, TLI = 0.90, and RMSEA = 0.05, Δχ^2^ = 137.968, Δ*df* = 3, *p* < 0.01) and temporary agency workers (χ^2^ = 729.721, *df* = 344, CFI = 0.92, TLI = 0.90, and RMSEA = 0.05, Δχ^2^ = 27.561, Δ*df* = 3, *p* < 0.01).

**TABLE 4:** Fit indices for the hypothesized model and alternative model.

	***X${}^{2}$***	***Df***	***p***	***ΔX${}^{2}$/df***	***IFI***	***TLI***	***CFI***	***RMSEA***	***AIC***	***BCC***
M1 (PE)	838.782	341	0.000	X	0.93	0.92	0.93	0.04	1084.782	1094.429
M2 (PE)	976.750	344	0.000	137.968/3**	0.92	0.90	0.92	0.05	1216.750	1226.162
M1 (TAW)	702.160	341	0.000	X	0.92	0.91	0.92	0.05	948.160	963.039
M2 (TAW)	729.721	344	0.000	27.561/3**	0.92	0.90	0.92	0.05	969.721	984.237

PE, permanent employees (*N* = 769) and TAW, temporary agency workers (*N* = 527). ** *p* < 0.01.

## Discussion

Being employable is of key importance for employees in the contemporary work environment, especially now that workers are less likely to experience job security, as well as the increased emphasis on employee flexibility and mobility (Muffels, 2008). To overcome these challenges in the labor market, it has become increasingly relevant for employees to take a proactive stance toward both their job and career development (Crant, 2000). In this article, we aimed to gain more insight in the relationship between both permanent and temporary workers’ job crafting behaviors and their perceptions of their chances to gain new employment in the future. Additionally, we investigated whether organizations can facilitate these proactive initiatives by creating a psychologically safe workplace. Finally, and due to the current shift to organizations using more temporary work arrangements, we examined the indirect effect between psychological safety and perceived employability trough job crafting behaviors.

Our study indicates that in particular for permanent employees, psychological safety is an important organizational factor associated with job crafting behaviors, as it was found to be positively related to increasing structural and social job resources, as well as increasing challenging job demands. These findings are consistent with previous research showing a link between psychological safety and proactive work behaviors, including voice and personal initiative (Baer and Frese, 2003; Detert and Burris, 2007; Ghitulescu, 2007). Interestingly, we found a negative relationship between psychological safety and decreasing hindering job demands in both groups of workers, suggesting that employees who feel psychologically safe in their organization are less likely to decrease their hindering job demands. Based on social exchange theory (Cropanzano and Mitchell, 2005), an alternative explanation of the negative relationship between psychological safety and decreasing hindering job demands could be that employees who feel psychologically safe within an organization, may feel inclined to do something back so that the relationship is equitable. By reducing their hindering job demands and thereby withdrawing from certain work tasks, employees may feel that they do not reciprocate the organization, and therefore may decide to refrain from such behaviors (Wayne et al., 1997).

Moreover, our findings reveal that for temporary workers, feeling psychologically safe is unrelated to increasing structural and social job resources, as well as challenging job demands, but similar to the permanent workers, feeling safe psychologically related negatively to decreasing hindering job demands. Concerning these notable differences between agency workers and permanent employees in the psychological safety – job crafting relationship, it could be the case, based on expectancy theory (Vroom, 1964, 2005), that workers engage in job crafting behaviors after weighing the anticipated costs and benefits of their behavior. Our results indicate that these costs may be more impactful for permanent employees, meaning that job crafting can yield both greater rewards as well as repercussions compared to agency workers. As such, for employees with a permanent position, psychological safety seems to play a more profound role as an instigator of proactive behaviors. Interestingly, temporary workers reported significantly lower levels of both psychological safety and all job crafting behaviors, with the exception of increasing structural job resources. In line with these findings, Han et al. (2009) found that temporary employees felt less empowered by their organization compared to their colleagues who held a permanent position within the same organization. In turn, a lack of empowerment is generally linked to lower levels of employee proactivity and innovative behaviors (Kirkman and Rosen, 1999; Spreitzer et al., 1999).

The present study’s findings also encourage further investigation of job crafting as a strategy for different types of workers, since our results suggest that for permanent, and temporary workers different job crafting strategies are related to employability. First, increasing structural job resources was only positively associated with perceived employability for agency workers and not for permanent employees, indicating that for temporary workers, especially proactively seeking out new learning experiences and investing in professional development is related to the individual perception of one’s employability. In line with our reasoning, it seems that for temporary workers, crafting a professional skill set and responsibility is more relevant in terms of feeling employable, potentially because these skills are easier to transfer to a new job compared to for instance more specific new task experiences. Additionally and in contrast to our expectations, increasing social job resources was unrelated to perceived employability for both types of workers. It could be that proactive employees who seek out feedback, advice, and support from colleagues and supervisors may be offered and gain more job-specific guidance and skills, which in turn translates into more perceived internal compared to external employability. In addition, internal colleagues may be less knowledgeable about the external labor market. Third, seeking challenges within the current job was related to increased perceptions of employability, but only for permanent workers. A possible explanation for this finding could be that in terms of feeling employable outside of the current organization, permanent workers could benefit more from actual and more tangible new task experiences, which might also be transferrable to a new job, and organization. Last, as expected, decreasing hindering job demands was negatively associated with employability for the same group, confirming our notion that employees may perceive the necessity to reduce their job demands as a sign of a lack of career-related know-how. In particular, for temporary workers, crafting job demands was found to be unrelated to feeling employable. This finding is in line with Hakanen et al. (2006), who stated that job demands are relatively stable aspects of a job, that require time and effort in order to change. Due to the limited time frame for temporary workers to perform within an organization, crafting job demands may distract them from their core job tasks and as such, does not contribute to a sense of being employable.

### Theoretical Implications

A first contribution of this article is that, next to the common approach of studying work behaviors among permanent employees, we studied job crafting behaviors in a new and contemporary organizational context, namely that of the temporary workforce. Research on job crafting has grown rapidly in the past decade. However, until now, research has mainly focused on job crafting among employees who hold a permanent position. Although our findings indicate that job crafting is associated with employability for both types of workers, it appears that for temporary agency workers the degree to which they feel safe has less impact on their proactive initiatives compared to permanent employees. This raises questions concerning the antecedents of job crafting for the temporary workforce. It could be that more direct forms of organizational involvement, such as management support and leadership, are better predictors of job crafting among temporary workers. This notion is supported by Wang et al. (2017), who found that in particular for employees with lower levels of organizational identification, transformational leadership was strongly associated with job crafting behaviors. This is an interesting finding in light of this study. Because temporary workers often report lower levels of organizational commitment (Foote, 2004), it could be that an active and empowering leadership style inspires more proactive behaviors among these workers compared to more passive beliefs about the organizational environment (i.e., psychological safety). Moreover, our findings also revealed that agency workers experienced less psychological safety compared to permanent workers and engaged in fewer job crafting behaviors. This is a worrying finding in itself, because compared to permanent employees, temporary workers have less access to formal, and informal training programs provided by their place of work (Forrier and Sels, 2003). Taken together, it seems that due to these challenging working conditions, it is even more important for temporary agency workers to implement proactive strategies such as job crafting to increase their employability.

Second, we aimed to answer the call by Oldham and Fried (2016) for more research on the contextual factors of individual job crafting. By taking into account the extent to which workers felt safe to engage in proactive behaviors, we revealed that the expectations employees have concerning the outcome of their initiatives are directly related to actual job crafting behaviors. This finding underlines the importance of an organizational climate that facilitates proactive work behaviors (see also: Baer and Frese, 2003).

Last, we have strived to create a bridge between the literature on job design and career theory. Most studies on proactive work behavior and job crafting have solely focused on job-related outcomes. However, our findings indicate that the display of proactive behaviors on the job, and in particular crafting autonomy, variety, developmental opportunities, and seeking out new projects, are linked to a heightened sense of being employable outside the current organization (Brenninkmeijer and Hekkert-Koning, 2015; Akkermans and Tims, 2017). Hence, it seems that by accumulating both job resources and challenging job demands, employees are able to not only optimize important work-related outcomes, but also can use these obtained abilities and knowledge to the advantage in terms of their career progress.

### Limitations

A first limitation of the study is that we used a cross-sectional research design. Although we gained access to two large groups of temporary and permanent employees, we were not able to follow them over time. It could be that some of the proposed relationships are in reality reversed or reciprocal, for example, when employees with high levels of perceived employability feel more empowered to engage in job crafting behaviors. The findings of this study should therefore be replicated in future research with a longitudinal study design.

Furthermore, the use of self-reports can lead to common method bias (Podsakoff et al., 2003). However, we obtained different results for both groups and in addition to the satisfactory fit of the measurement model, we performed Harman’s one-factor test, which showed that the variance in our data could not be attributed to a single factor. Together, the statistical differences between the two groups and the one-factor test, indicate that common method bias is not a major issue in this study. In addition, the Cronbach’s alpha for the psychological safety scale, was relatively low in both groups of employees. However, according to Hair et al. (2006), Cronbach’s alpha values between 0.6 and 0.7 are acceptable, in particular when a scale does not consist of many items.

Finally, this sample was obtained within the same temporary agency bureau and agency workers – similarly to the permanent employees – were deployed in various organizations and occupational sectors. In accordance with this sample of temporary workers, we aimed to obtain comparable data among permanent employees, also working for various organizations and sectors. This latter sample contained student-recruited employees. However, Demerouti and Rispens (2014) argue and demonstrate that student-recruited samples do no differ from non-student-recruited samples in terms of generalizability and validity. To further generalize our findings, it would be of interest to investigate how temporary workers perceive their work environment compared to permanent employees working within the same organizational setting. In addition, it would be interesting to see whether our findings are also applicable to other forms of non-standard employment arrangements, such as direct-hiring and self-employment.

### Practical Implications

Our findings have several implications for managers and HR practitioners. First, the positive relationship between psychological safety and increasing structural job resources and challenging job demands, indicates that organizations may play an important role in providing an environment in which proactive work behaviors are encouraged, especially for permanent employees. In particular, now that accountability for career advancement and planning is shifting from the organization toward the individual, managers should aim to shape a workplace in which employees are able to develop themselves professionally and can act upon proactive intentions. In addition, it is likely that feeling employable is not the only outcome affected by job crafting initiatives. Other important individual and organizational outcomes, such as work engagement, job performance, well-being, and career success are also found to be directly influenced by employees’ job crafting behaviors (Bakker et al., 2012; Tims et al., 2013, 2015; Plomp et al., 2016; Akkermans and Tims, 2017). This further emphasizes the importance of an organizational climate and leadership style in which proactive work behaviors are facilitated and supported.

Finally, considering that temporary workers are faced with less job security compared to permanent employees, investing in career development programs and actively supporting proactive initiatives is likely to result in more employability, as well as positive attitudes and commitment toward the organization (Chambel and Sobral, 2011). This may, for instance, be achieved by stimulating employees to engage in learning programs and raising awareness how they can adapt their job so that it better fits their preferences and aligns with their career goals. Additionally, organizations could implement a job crafting intervention for both temporary and permanent employees, designed to learn how to acquire and build job resources, and seek out challenging demands (Van Den Heuvel et al., 2015). This could help workers to become better managers of their work and career.

## Author Contributions

JP designed the study, collected, and analyzed the data. All authors listed have made a substantial and equal contribution to the draft of the manuscript.

## Conflict of Interest Statement

The authors declare that the research was conducted in the absence of any commercial or financial relationships that could be construed as a potential conflict of interest.
